# Timing and other factors influencing shortterm outcomes of endoscopic transpapillary gallbladder drainage for acute cholecystitis: a retrospective study

**DOI:** 10.1186/s12876-025-04027-2

**Published:** 2025-07-01

**Authors:** Fumitaka Niiya, Naoki Tamai, Masataka Yamawaki, Jun Noda, Tetsushi Azami, Yuichi Takano, Fumiya Nishimoto, Masatsugu Nagahama

**Affiliations:** Division of Gastroenterology, Department of Internal Medicine, Showa University Fujigaoka Hospital, 1-30 Fujigaoka, Aoba-ku, Yokohama, Kanagawa Japan

**Keywords:** Acute cholecystitis, Endoscopic transpapillary gallbladder drainage, Laparoscopic cholecystectomy, Postoperative adverse events

## Abstract

**Background:**

Endoscopic transpapillary gallbladder drainage (ETGBD) is used to manage acute cholecystitis (AC) in patients in whom surgery is contraindicated. However, ETGBD is considered challenging, has comparatively lower success rates, and is associated with severe adverse events (AEs). Only a few studies have examined the procedural and anatomical factors that affect the success of technical ETGBD. This study identified predictive factors for ETGBD in AC to improve success rates and minimize AEs.

**Methods:**

Patients treated with ETGBD for AC were assessed. Factors associated with technical failure were analyzed based on the interval from symptom onset to ETGBD, the presence of cystic duct stones, and cystic duct diameter.

**Results:**

Among 68 patients, the technical success and AE rates were 64.7% and 19.1%, respectively (cystic duct perforation, 8.8%; pancreatitis, 5.9%). Multivariate analysis revealed that early ETGBD and stone impaction in the cystic duct were significantly associated with technical ETGBD failure. Subgroup analysis demonstrated that early ETGBD was associated with a significantly higher risk of cystic duct perforation and a lower success rate than elective interventions. Thus, elective ETGBD may enhance procedural success and reduce the risk of cystic duct perforations.

**Conclusion:**

Elective ETGBD may be considered in cases in which ETGBD is anticipated to be challenging.

## Background

Laparoscopic cholecystectomy is an established standard treatment for acute cholecystitis (AC) [1–3]. However, in some patients, surgical intervention may be contraindicated because of the presence of comorbid conditions. In such patients, nonsurgical options for gallbladder drainage, such as percutaneous transhepatic gallbladder drainage (PTGBD), percutaneous transhepatic gallbladder aspiration (PTGBA), endoscopic transpapillary gallbladder drainage (ETGBD), and endoscopic ultrasound-guided gallbladder drainage (EUS-GBD), can be used to effectively decompress the gallbladder. The revised 2018 Tokyo Guidelines recommend PTGBD as a primary nonsurgical alternative for the management of AC [2]. However, significant recurrence rates of cholecystitis (22–47%) have been reported in patients who do not undergo cholecystectomy after the removal of the percutaneous catheter [4, 5]. Therefore, the use of an ETGBD as an internal stent is an important treatment approach.

ETGBD is a method used for gallbladder drainage in the treatment of AC [6–9]. However, ETGBD is considerably challenging to perform, with reported success rates ranging between 64% and 100% [10]. Moreover, there is a risk of adverse events (AEs) associated with endoscopic retrograde cholangiopancreatography (ERCP) and the likelihood of perforation of the cystic duct during ETGBD. Therefore, it is crucial to determine how to increase the success rate of ETGBD. However, only a few studies have examined procedural and anatomical factors affecting the technical success of this procedure. Additionally, preoperative assessment of these anatomical factors is often challenging. Thus, it is important to identify the factors that the operator can control.

Conversely, regarding the timing of surgery for AC, surgeries performed later rather than early in the course of treatment resulted in fewer postoperative AEs and a reduced need for conversion to open surgery due to attenuation of inflammation and adhesions [11, 12]. Similarly, in the context of ETGBD, tissue weakening due to inflammation and the presence of adhesions could potentially contribute to procedural failure and AEs. Therefore, this study aimed to investigate the factors influencing technical failure, including the timing of ETGBD.

## Methods

### Study population

This single-center retrospective analysis focused on patients who underwent ETGBD for AC based on clinical symptoms, laboratory data, and imaging studies, in accordance with the 2018 Tokyo Guidelines (TG18), between January 2016 and December 2023. The exclusion criteria were as follows: (1) patients with surgically altered gastrointestinal anatomy, except for those who underwent Billroth I reconstruction, and (2) patients with pancreaticobiliary malignancies. Ethical compliance was ensured in accordance with the guidelines set by the Institutional Review Board of the hospital, and the study was conducted in accordance with the tenets of the Declaration of Helsinki.

### ETGBD procedure

All the patients included in this study underwent ERCP under sedation. Standard duodenoscopes (JF-260 V, TJF-Q260V, or TJF-Q290V; Olympus Medical Systems, Tokyo, Japan) were used to perform ETGBD. After successful cannulation of the bile duct, cholangiography was performed to identify the location of the cystic duct. Endoscopic stone extraction was performed in patients with common bile duct stones. A 0.025-inch guidewire (VisiGlide2; Olympus Medical Systems) and ERCP catheter (PR-V614M, Olympus Medical Systems) were used to seek and advance through the cystic duct into the gallbladder. The guidewire and cannula were then advanced into the cystic duct, and a contrast medium was injected to evaluate the structure of the cystic duct, which was dilated using a 6-Fr Soehendra^®^ dilation catheter (Cook Medical, Bloomington, IN, USA) if necessary. Subsequently, an ERCP catheter was inserted over the guidewire in the gallbladder for bile aspiration and saline irrigation. The procedure was concluded with the placement of a plastic stent (5-Fr 10-cm IYO-stent; Gadelius Medical, Tokyo, Japan) or a 6-Fr endoscopic nasobiliary drainage (ENBD) tube, as decided by the endoscopist, based on the specific requirements of each case.

### Study outcomes and definitions

Data on the following parameters were collected: patient background, disease severity, interval from symptom onset to ETGBD, diameter of the common bile duct, presence or absence of cystic duct stones, bifurcation of the cystic duct, and cystic duct perforation. The primary outcomes were the technical success rate and AEs.

**Fig. 1:**
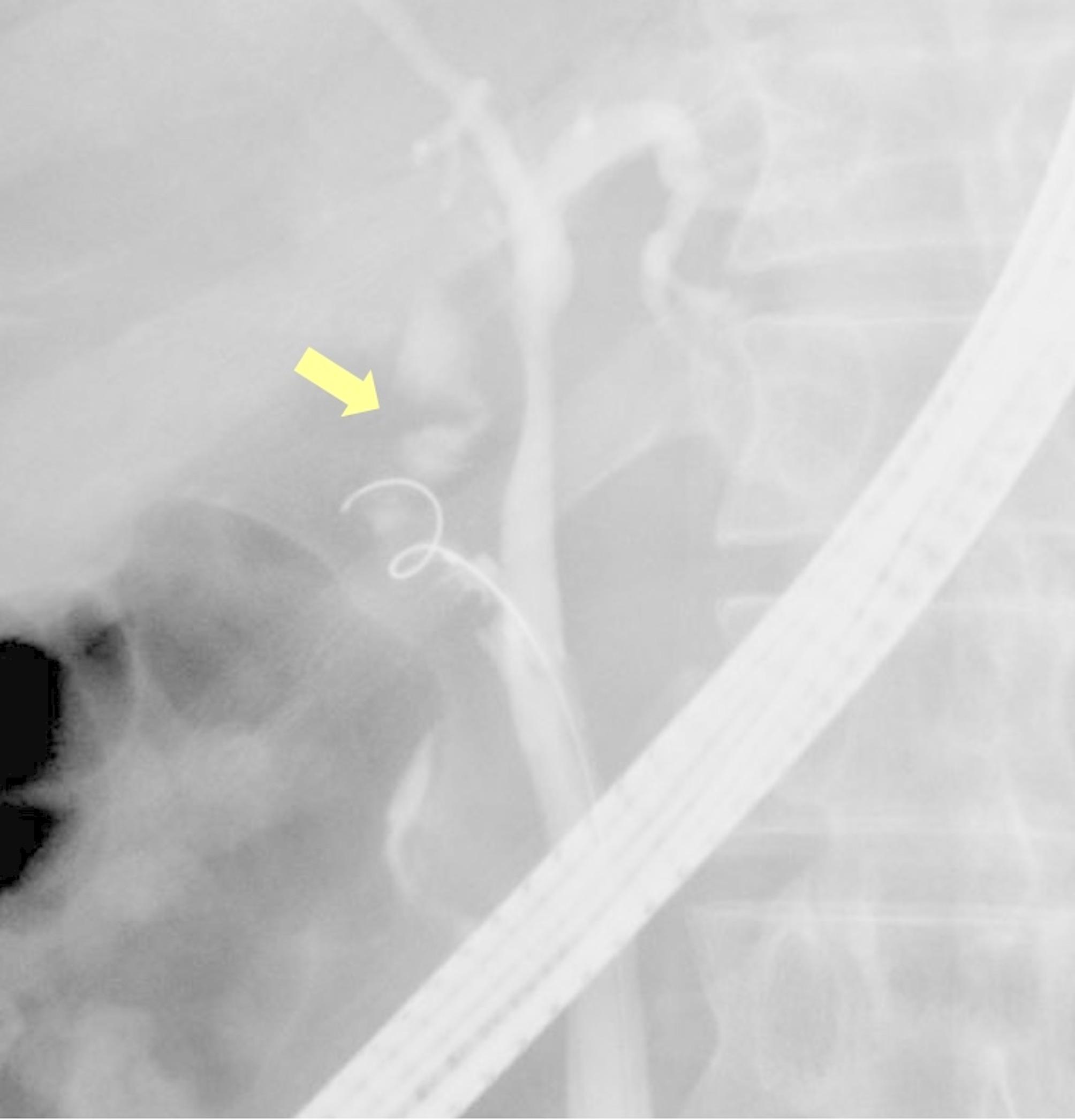
Fluoroscopic images of cystic duct perforation. Yellow head shows leakage of the contrast medium into the peritoneal cavity from the cystic duct lumen

The severity of AC was classified according to the 2018 Tokyo Guidelines. Cystic duct perforation was identified using device dislocation, such as a guidewire or cannula, or by fluoroscopically confirming the leakage of contrast medium from the cystic duct lumen into the peritoneal cavity (Fig. 1). The interval between symptom onset and ETGBD was defined as the number of days between the symptom onset and ETGBD. The technical success of ETGBD was determined by the successful placement of a stent or nasobiliary drainage catheter in the gallbladder through the cystic duct. AEs were classified based on the Tokyo Criteria [13], and their severity was graded according to the American Society of Gastrointestinal Endoscopy lexicon guidelines [14].

The final assessment of the direction and location of the cystic duct was determined based on findings from ERCP, computed tomography (CT), or magnetic resonance cholangiopancreatography (MRCP).

### Statistical analysis

Continuous variables are presented as medians and interquartile ranges and compared using the Mann–Whitney *U* test. Categorical variables are presented as proportions and compared using Fisher’s exact test. Multivariable logistic regression analyses were performed to identify predictive factors for the technical failure of ETGBD. The following variables were evaluated to elucidate the predictive factors: the presence of stone impaction in the cystic duct (with vs. without), common bile duct diameter (*n* ≤ 7 mm vs. > 7 mm), direction of the cystic duct (right caudal vs. right cranial + left cranial), contrast cystic duct (with vs. without), and interval from symptom onset to ETGBD (early vs. elective). The cut-off value for common bile duct diameter was set at 7 mm, based on a previous study that reported that a common bile duct diameter ≥ 7 mm contributed to the technical failure of ETGBD [15]. Receiver operating characteristic (ROC) curves for the interval from symptom onset to ETGBD for technical failure of ETGBD were plotted, and the accuracy of the area under the curve (AUC) was evaluated. The cut-off time for the interval from symptom onset to ETGBD (early and elective) was determined using the ROC curve analysis. Statistical significance was set at *p* < 0.05. All analyses were performed using R version 3.4.1 (The R Foundation for Statistical Computing, Vienna, Austria).

## Results

### Patient characteristics

The patient characteristics are presented in Table 1. This study included a cohort of 68 patients who underwent ETGBD between January 2016 and December 2023. The median age of the study population was 75 years (interquartile range, 67.5–82.5 years. Most of the study participants were male (79.4%). Classification of cholecystitis severity, adhering to the TG18, identified 4.4% of cases as mild, 77.9% as moderate, and 17.6% as severe. The median interval from symptom onset to ETGBD was seven days. The median cystic duct diameter was 3 mm (interquartile range, 2.6–4 mm, and stones were impacted in the cystic duct in 13.2% of cases. The median diameter of the common bile duct was 8 mm with an interquartile range of 6–9 mm.

**Table 1:** Patient characteristics

	ETGBD (*n* = 68)
Age, median, years (IQR)	75 (67.5–82.5)
Sex, male, n (%)	54 (79.4)
Severity of cholecystitis, n (%)	
Mild	3 (4.4)
Moderate	53 (77.9)
Severe	12 (17.6)
The interval from symptom onset to ETGBD, days (IQR)	7 (2.5–16)
Presence of CBD stone, n (%)	33 (48.5)
Cystic duct diameter, mm (IQR)	3 (2.6–4)
Stone impaction in the cystic duct	9 (13.2)
CBD diameter, median, mm (IQR)	8 (6–9)

CBD, common bile duct; ETGBD, endoscopic transpapillary gallbladder drainage; IQR, interquartile range

### Outcomes of ETGBD

The outcomes of the ETGBD are listed in Table 2. In the cohort of 68 patients who underwent ETGBD, the technical success rate was 64.7%. Endoscopic nasogalbladder drainage (ENGBD), defined as ENBD to the gallbladder, was performed in 11.8% (8 cases) and endoscopic gallbladder stenting (EGBS) in 52.9% (36 cases). The removal of common bile duct stones was performed in 41.2% (28 cases). Non-contrast cystic ducts were noted in 19.1% of the patients (13 cases). Most cystic ducts (94.1%, 64 patients) were oriented in the right caudal direction, with only a minority in the right or left cranial orientation or unknown (two cases).

**Table 2:** Endoscopic procedure

	ETGBD
	*n* = 68
Technical success, n (%)	44 (64.7)
ENGBD	8
EGBS	36
CBD stone removal, n (%)	28 (41.2)
Non-contrast cystic duct, n (%)	13 (19.1)
The direction of the cystic duct, n	
Right caudal/right cranial + left cranial/unknown	64/2/2

CBD, common bile duct; EGBS, endoscopic gallbladder stenting; ENGBD, endoscopic nasobiliary gallbladder drainage

In cases in which ETGBD was unsuccessful (*n* = 24), technical failure occurred because of several factors. First, in 10 cases, severe inflammation and edema made it difficult to identify the cystic duct. Second, in eight cases, the passage of the guidewire was obstructed by tortuosity, stenosis, or stones within the cystic duct. Finally, in six cases, the passage of the stent was hindered because of the same issues of tortuosity, stenosis, or stones.

Moreover, in failure cases, alternative management strategies were employed. PTGBD, PTGBA, and ENBD were performed in 15, 2, and 5 patients, respectively. Additionally, two patients who were unfit for invasive procedures received conservative management with antibiotics.

### Adverse events

Table 3 summarizes the AEs associated with ETGBD. AEs were documented in 19.1% (13 cases). Specific AEs included cystic duct perforation in 8.8% (6 cases), pancreatitis in 5.9% (4 cases), and cholangitis in 5.9% (4 cases). Bleeding was not observed.

**Table 3:** Adverse events

	ETGBD *n* = 68
Adverse events, n (%)	13 (19.1)
Cystic duct perforation	6 (8.8)
Pancreatitis	4 (5.9)
Cholangitis	3 (4.4)
Bleeding	0

### Predictive factors for technical failure of ETGBD

**Fig. 2:**
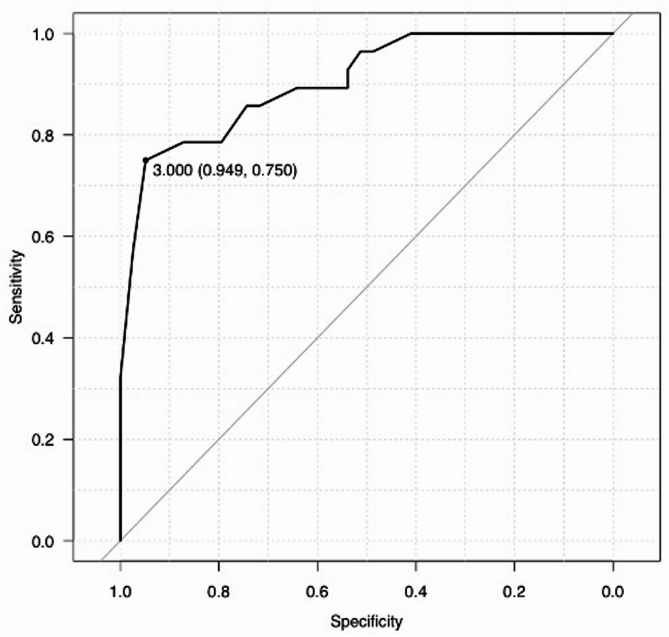
Using the receiver operating characteristic (ROC) curve for the interval from symptom onset to endoscopic transpapillary gallbladder drainage (ETGBD) for technical success. A cut-off value of three days (using the Youden index [sensitivity + specificity– 1]) with an area under the curve (AUC) of 0.91 (95% confidence interval [CI] 0.83–0.98) was determined

Using the ROC curve of the interval from symptom onset to ETGBD, the cut-off value and AUC for successful ETGBD were calculated to be 3.0 days and 0.91 (95% CI, 0.83–0.98) (Fig. 2). Table 4 shows the predictive factors for technical failure of ETGBD. Multivariate analysis revealed that early procedure (OR, 44; 95% CI, 6.1–317; *p* < 0.01) and presence of stone impaction in the cystic duct (OR, 13.5; 95% CI, 1.41–129; *p* < 0.02) were independent predictive factors for technical failure of ETGBD.

**Table 4:** Predictive factors for technical failure of ETGBD

			OR [95% CI]		*p* value	
Early ETGBD procedure			44 [6.1–317]		< 0.01	
The presence of stone impaction in the cystic duct			13.5 [1.41–129]		0.02	
CBD diameter (> 8 mm)			5.92 [0.97–35.9]		0.05	
The direction of the cystic duct			6.52 [0.28–152		0.2	
	Right caudal			6.52 [0.28–152]		0.24
Cystic duct without contrast,			1.15 [0.14–9.4]		0.9	

CBD, common bile duct; ETGBD, endoscopic transpapillary gallbladder drainage

### Comparison of outcomes of early and elective ETGBD

Table 5 shows the subgroup analysis of ETGBD outcomes; among the 45 patients in the elective group, percutaneous drainage was more frequent before ETGBD, with 80% (36 cases) having undergone PTGBD and only 2.2% (1 case) having PTGBA, compared to 4.3% (1 case) and 8.7% (2 cases) in the early group, respectively (*p* < 0.01 for PTGBD, *p* = 0.26 for PTGBA).

**Table 5:** Comparison of the outcomes of early and elective ETGBD

	Early group	Elective group	*p* value
	*n* = 23	*n* = 45	
Percutaneous drainage prior to ETGBD, n (%)			
PTGBD	1 (4.3)	36 (80)	< 0.01
PTGBA	2 (8.7)	1 (2.2)	0.26
Technical success, n (%)	12 (52.2)	32 (71.1)	0.18
Adverse events, n (%)			
Cystic duct perforation	5 (21.7)	1 (2.2)	0.01
Pancreatitis	0	4 (8.8)	0.29
Cholangitis	2 (8.7)	0	0.11

ETGBD, endoscopic nasobiliary gallbladder drainage; PTGBD, percutaneous transhepatic gallbladder drainage; PTGBA, percutaneous transhepatic gallbladder aspiration

Technical success was achieved in 71.1% (36 cases) of the elective group and 52.2% (12 cases) of the early group (*p* = 0.18). AEs varied significantly, with cystic duct perforation occurring in 2.2% (1 case) of the elective group compared to 21.7% (5 cases) of the early group (*p* = 0.01). Pancreatitis was observed in 8.8% (4 cases) of patients in the elective group, while pancreatitis was not observed in the early group (*p* = 0.29). Conversely, cholangitis did not occur in the elective group but was observed in 8.7% (2 cases) in the early group (*p* = 0.11).

## Discussion

To the best of our knowledge, this is the first study to focus on the timing of ETGBD. Multivariate analysis revealed that early ETGBD was a significant predictor of technical failure. Notably, the early group had a significantly higher incidence of cystic duct perforation compared to the elective group.

Several studies have investigated the factors contributing to the technical failure of ETGBD in the context of AC [15–17]. It has been suggested that the anatomy of the cystic duct bifurcation may contribute to unsuccessful outcomes [16]. Moreover, cystic duct perforation has been reported to be a factor in unsuccessful ETGBD [17]. However, preoperative identification of the cystic duct bifurcation anatomy is challenging. Sato et al. [18] reported that identification of cystic duct bifurcation using CT or MRCP is possible in only 60% of cases. Similarly, cystic duct perforation is a severe AE of ETGBD and is not a pre-procedural predictive factor. This study focused on the timing of ETGBD and early ETGBD, and the presence of cystic duct stones was found to be significantly associated with technical failure of ETGBD.

Elective ETGBD may not only enhance success rates but also potentially reduce AEs. Studies have suggested that patients operated on for AC after a delay of 72 h (3 days) post-onset exhibit fewer postoperative AEs and shorter hospital stays than those operated on within 72 h [19]. This could be attributed to early intervention, which led to lower success rates and increased AEs due to inflammation-induced tissue vulnerability and adhesions. Furthermore, Sofuni and Itoi [20] reported that cystic duct perforation, a critical AE of ETGBD, is correlated with edematous inflammatory changes in the cystic duct. In our subgroup analysis, early ETGBD had lower technical success rates and a significantly higher incidence of cystic duct perforations. Elective ETGBD increases the likelihood of success and provides a safer treatment approach by minimizing AEs. However, in the elective group, 80% of patients had previously undergone PTGBD. The use of PTGBD to suppress inflammation and adhesion progression before surgery has frequently been reported in the literature, suggesting that PTGBD before ETGBD could be beneficial [21].

Therefore, based on the findings of this study, it is difficult to determine which approach (elective ETGBD or PTGBD) contributes more to improving technical success or reducing AEs. Nonetheless, in patients with moderate or severe acute cholecystitis, PTGBD is generally required prior to elective ETGBD. Therefore, we believe that our results offer valuable insights into clinical practice. However, prolonged hospital stay and a decline in activities of daily living may arise from PTGBD placement, underscoring the need for individualized treatment strategies based on the patient’s condition.

An additional and critical consideration is the long-term follow-up. Our study focused exclusively on short-term outcomes and did not evaluate the long-term outcomes. A previous report noted a recurrence rate of only 3.7% (1/27 patients) after ETGBD was performed following PTGBD, suggesting that this staged approach may offer a durable benefit [22]; however, these data are too limited to draw definitive conclusions. Therefore, larger studies with systematic, long-term follow-up are needed to establish the true clinical value of elective ETGBD.

The indications for PTGBD in patients undergoing antithrombotic therapy remain controversial. While TG18 recommends avoiding PTGBD in patients taking antithrombotic medication [2], the Society of Interventional Radiology advocates its use without discontinuing medication when there is a risk of thrombosis, leaving no consensus [23]. Based on our clinical experience, cases necessitating emergency ETGBD may not be as frequent as expected. Therefore, in patients in whom ETGBD is expected to be challenging or those at a higher risk of post-ERCP pancreatitis, proceeding with PTGBD while on antithrombotic therapy, followed by elective ETGBD, may be a prudent strategy.

In this study, the ETGBD success rate was slightly lower than that previously reported. A contributing factor to this finding may be the limited selection of devices available for use. Specifically, difficulty in identifying the cystic duct was encountered because of severe inflammation and edema in 41.6% of cases, and stent passage was hindered by tortuosity, stenosis, or stones in 25% of cases. Yoshida et al. [24] reported a significantly higher success rate with SpyGlass DS-assisted ETGBD than with the conventional methods, which could help in navigating and visualizing difficult cystic ducts. Ban et al. [25] documented the successful use of a Soehendra^®^ stent retriever to fragment stone impactions within the cystic duct and facilitate a successful ETGBD. The use of such devices may resolve the challenges encountered during ETGBD, and the inclusion of various devices in future studies may potentially improve the outcomes of ETGBD.

The introduction of lumen-apposing metal stents (LAMS) has made EUS-GBD a notable alternative to surgery [26]. A meta-analysis showed that EUS-GBD with LAMS achieved technical and clinical success rates of 100% and 85%, respectively [27]. Despite its effectiveness, the use of LAMS for gallbladder drainage has not been officially approved, and only a few experienced specialists perform EUS-GBD. Compared with EUS-GBD, ETGBD offers unique benefits, such as maintenance of natural anatomy and better long-term outcomes with stent placement [28]. Enhancing the technical success of ETGBD is crucial, making this research significant for advancing gallbladder drainage techniques. Treatment options should be selected according to the patient’s background and conditions. Further accumulation of cases and investigation of the long-term outcomes in future studies are warranted.

This study has several limitations that need consideration. First, this was a single-center retrospective study with a relatively small sample size, which may limit the generalizability of the findings and potentially limit their applicability to other facilities with different levels of expertise or variations in available endoscopic devices. Moreover, altered gastrointestinal anatomy cases were excluded; together with the predominance of moderate severity cases, these factors further limit the generalizability of our findings. The retrospective design could also have introduced bias, affecting data collection and interpretation. Second, in this study, the direction of the cystic duct was determined using ERCP, CT, and MRCP; however, in patients with AC, accurate evaluation of the cystic duct may have been challenging because of inflammation and edema. Third, because of the small sample size, it is possible that not all factors influencing technical success were adequately evaluated. Moreover, no statistically significant difference in technical success rate was observed between the early and elective ETGBD groups (52% vs. 71%; *p* = 0.18). A sample size calculation indicated that at least 113 patients per group would be required to reliably detect differences of this magnitude. Further investigations through multicenter or prospective studies are warranted. Additionally, the choice to dichotomize the timing at approximately three days (derived from an ROC analysis) could introduce bias. Therefore, we conducted a sensitivity analysis using the median as the cut-off value in a multivariable model and obtained similar results. We believe this additional assessment further supports the usefulness and robustness of our findings. Fifth, our technical success rate is relatively low compared to some previous reports, which may reflect higher case complexity, limited device selection, or operator learning curves—factors that we did not analyze in detail. Nonetheless, this is the first study to explore the optimal timing of ETGBD, providing valuable insights despite these limitations.

## Conclusions

Early ETGBD was identified as a significant risk factor for technical failure, particularly because of the higher incidence of cystic duct perforation. These findings suggest that elective ETGBD, especially when preceded by PTGBD, may offer a safer and more effective approach in patients with moderate or severe AC or when procedural difficulty is anticipated. However, the retrospective single-center design and small sample size limited the generalizability of our results. Prospective, multicenter studies with long-term follow-up are warranted to validate these findings and further define the optimal patient selection and timing for ETGBD.

## Data Availability

The datasets generated and/or analysed during the current study are not publicly available because they contain patient‐level clinical information. De-identified data may be obtained from the corresponding author upon reasonable request and with approval of the Showa University Fujigaoka Hospital Ethics Committee.

## Abbreviations

AC: Acute cholecystitis
AE: Adverse events
AUC: Area under the curve
CBD: Common bile duct
CT: Computed tomography
EGBS: Endoscopic gallbladder stenting
ENBD: Endoscopic nasobiliary drainage
ERCP: Endoscopic retrograde cholangiopancreatography
ENGBD: Endoscopic nasogallbladder drainage
ETGBD: Endoscopic transpapillary gallbladder drainage
LAMS: Lumen-apposing metal stents
MRCP: Magnetic resonance cholangiopancreatography
PTGBA: Percutaneous transhepatic gallbladder aspiration
PTGBD: Percutaneous transhepatic gallbladder drainage
ROC: Receiver operating characteristic
